# Supplementary figures and images for: A novel RAB5 binding site in human VPS34-CII that is likely the primordial site in eukaryotic evolution
Source: eLife. 2026 May 28;15:RP110040. doi: 10.7554/eLife.110040 (PMC13218723; doi:10.7554/eLife.110040)

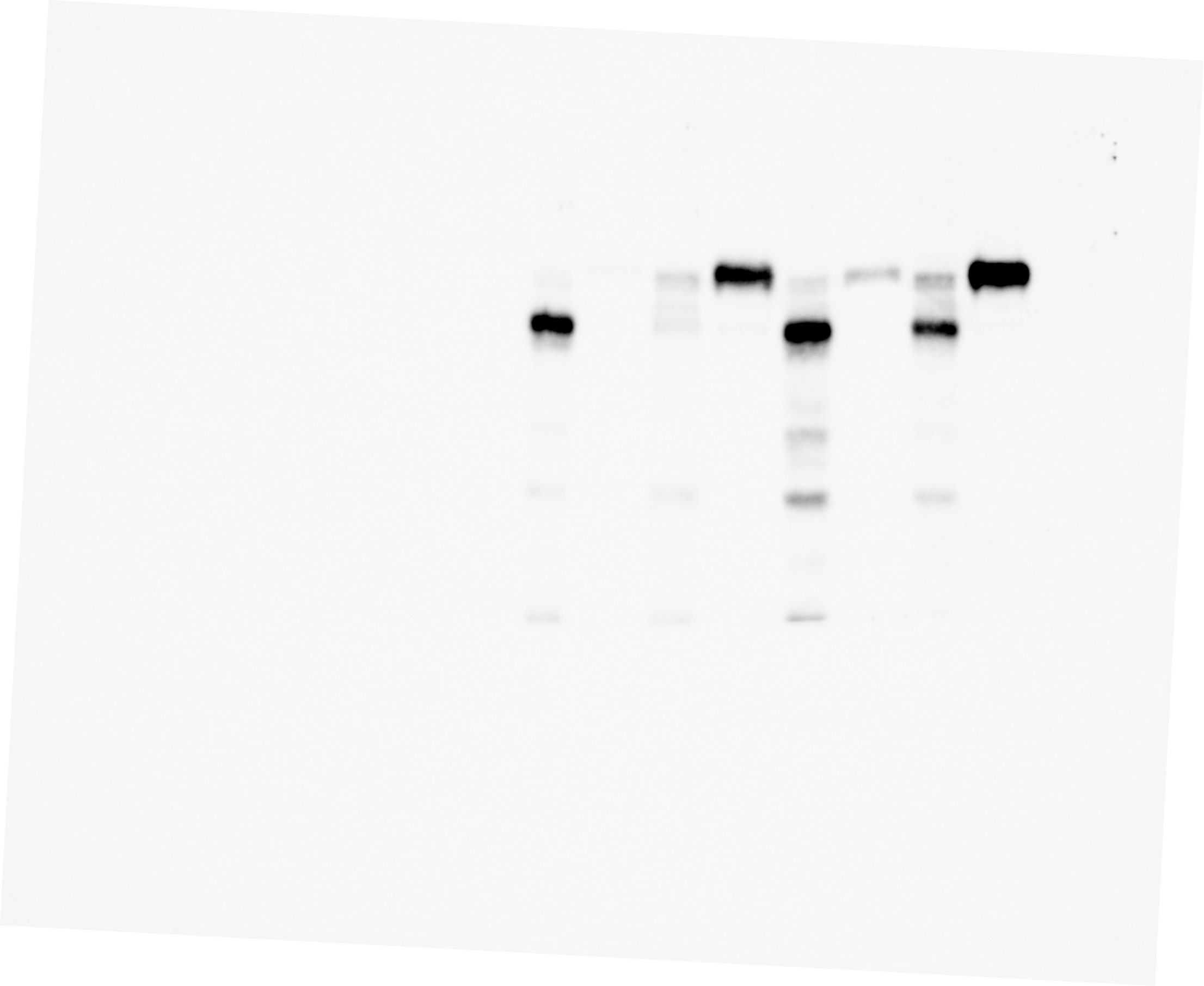

Supplement: Figure 2—source data 2. [file elife-110040-fig2-data2.zip › Figure2-source_data_1/anti-CPY.tif]

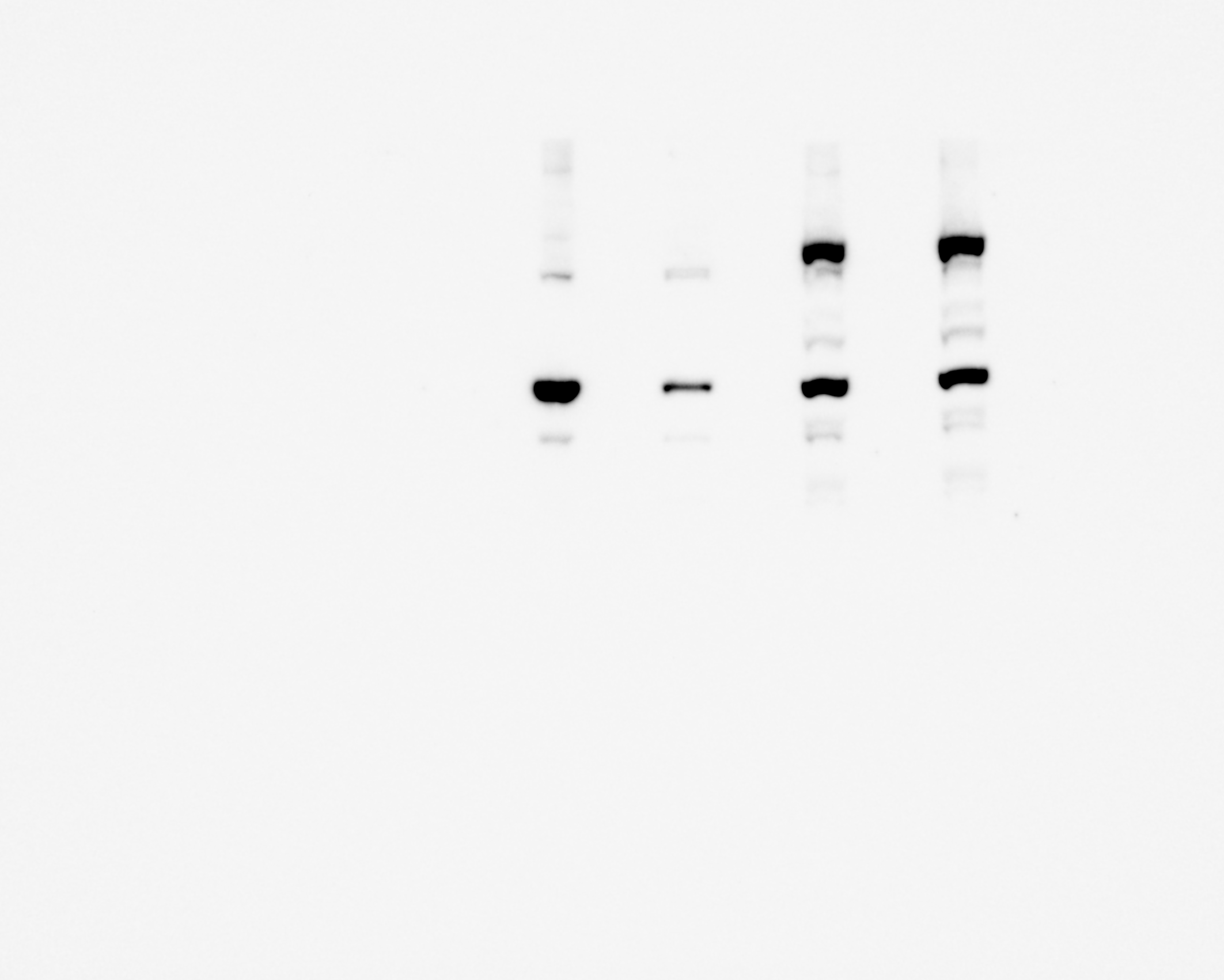

Supplement: Figure 2—source data 2. [file elife-110040-fig2-data2.zip › Figure2-source_data_1/anti-FLAG.tif]

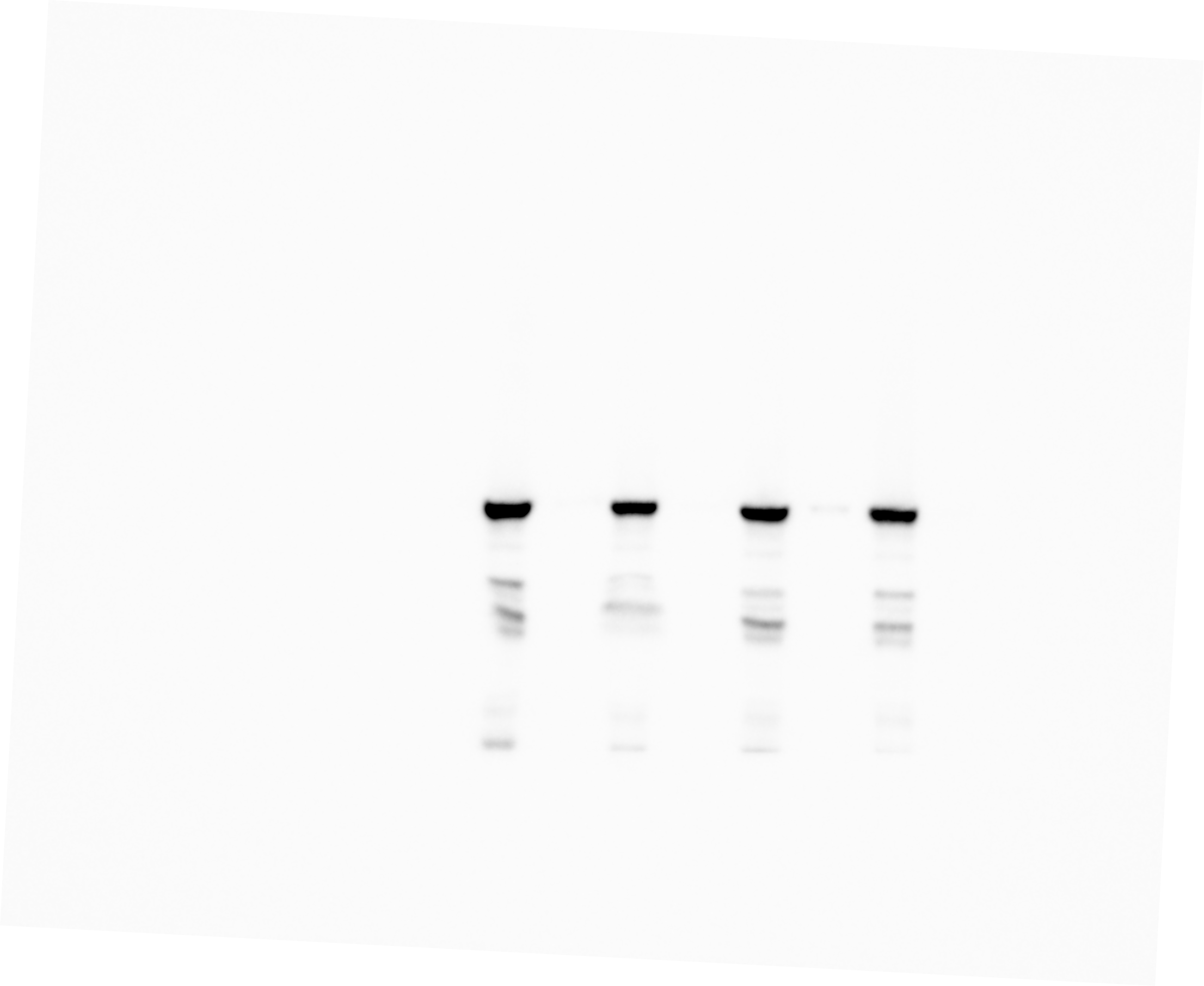

Supplement: Figure 2—source data 2. [file elife-110040-fig2-data2.zip › Figure2-source_data_1/anti-Pgk4.tif]

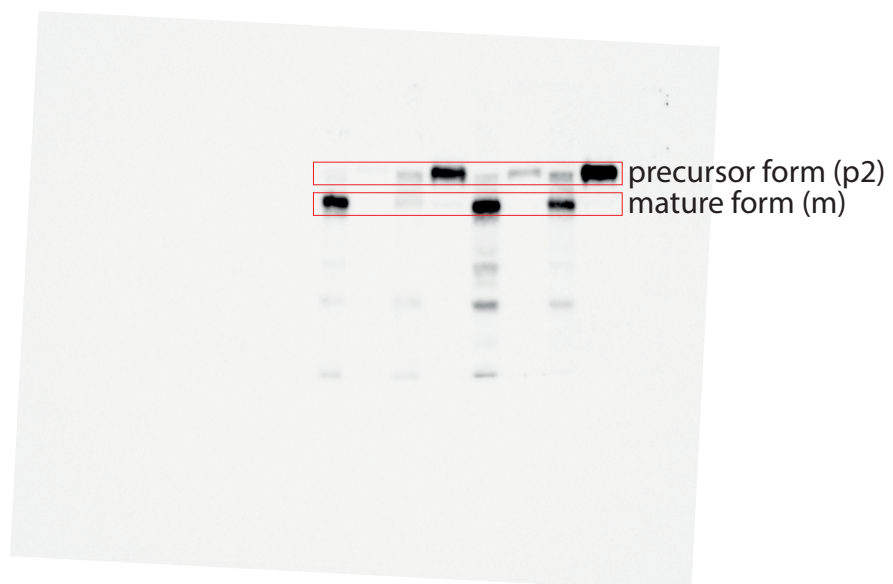

Supplement: Figure 2—source data 3. [file elife-110040-fig2-data3.zip › Figure2-source-data_2/anti-CPY.pdf]

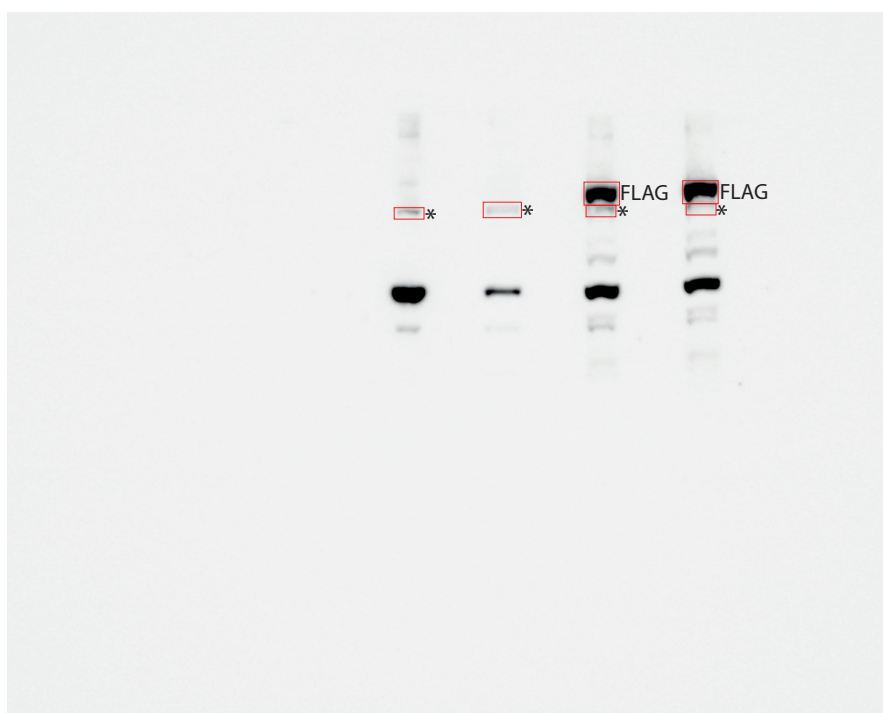

\* = non-specific cross-react

Supplement: Figure 2—source data 3. [file elife-110040-fig2-data3.zip › Figure2-source-data_2/anti-FLAG.pdf]

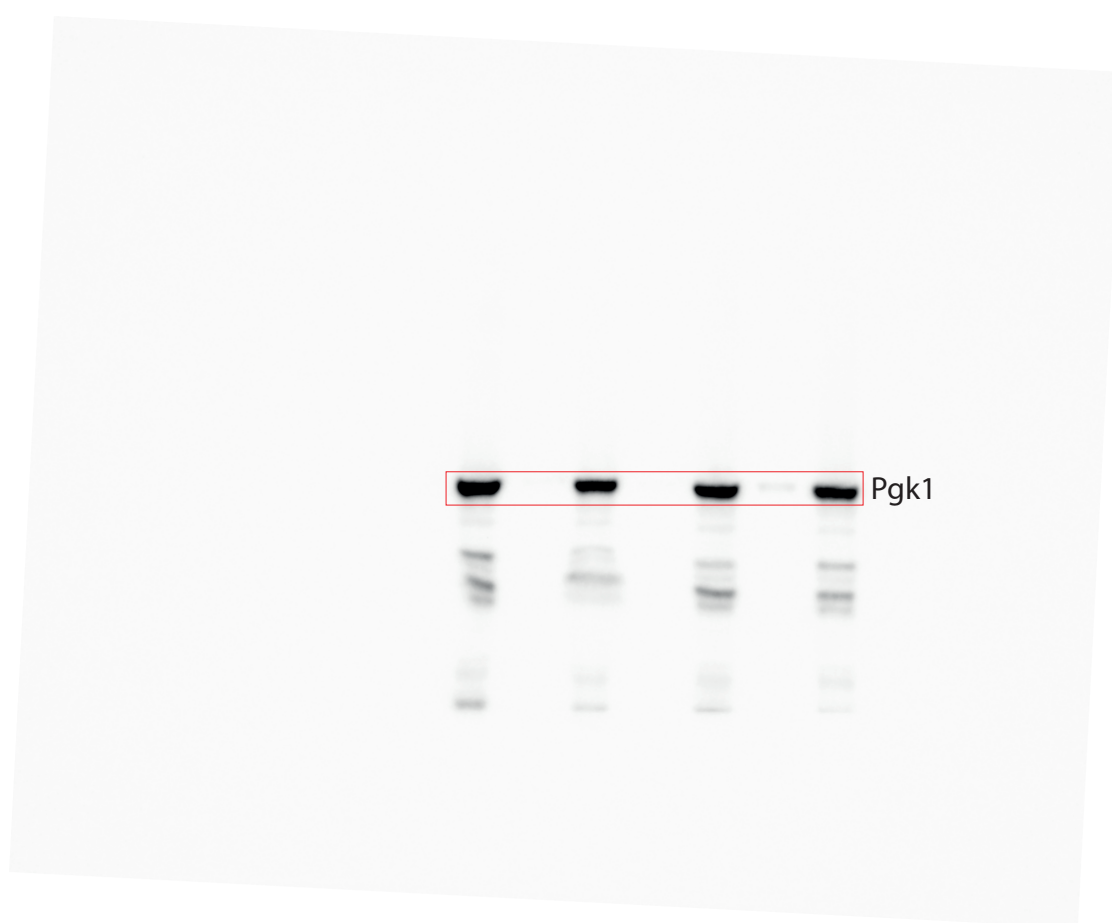

Supplement: Figure 2—source data 3. [file elife-110040-fig2-data3.zip › Figure2-source-data_2/anti-Pgk1.pdf]
